# Supplementary material for: Phosphorylation and Dephosphorylation of Beta-Amyloid Peptide in Model Cell Cultures: The Role of Cellular Protein Kinases and Phosphatases
Source: Life (Basel). 2023 Jan 4;13(1):147. doi: 10.3390/life13010147 (PMC9863727; doi:10.3390/life13010147)
Supplement: Supplementary file 1 [file life-13-00147-s001.zip › life-2032169-supplementary.pdf]

Supplementary Figures

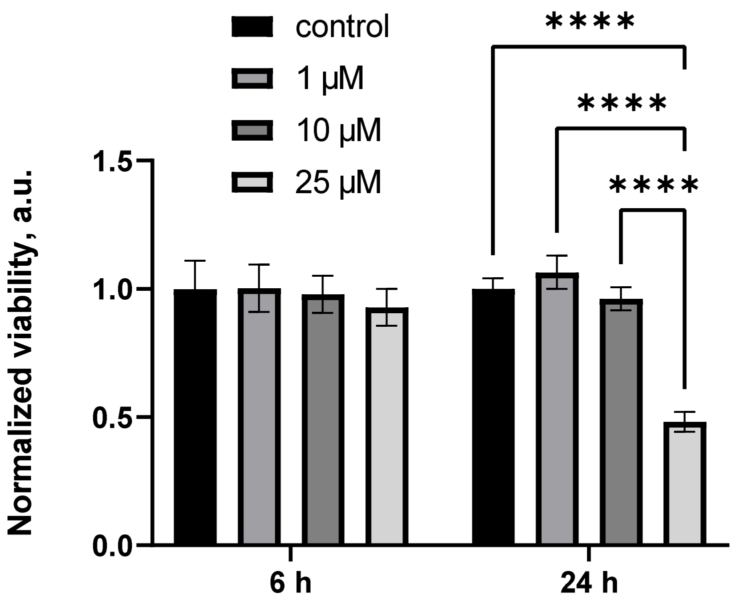

Figure S1. The toxicity of 1, 10 and 25 µM TBBz inhibitor for HEK293 cells at 6 and 24 hours of incubation measured with WST test. Data presented as means with SD normalized to respective controls in 6 and 24 hour groups. n=4, **** - $p<0,0001$ according to two-way ANOVA with post-hoc Tukey test.

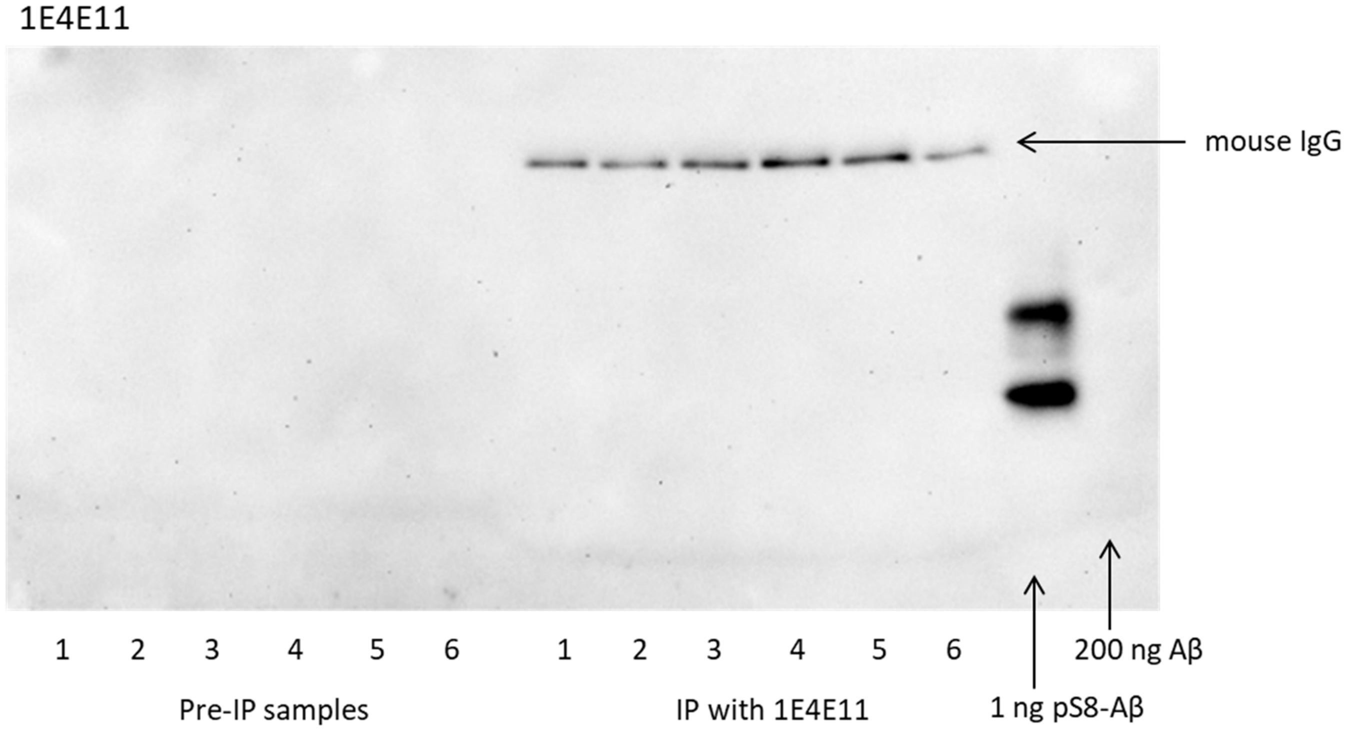

Figure S2. Phosphorylated Aβ in cell lysate of bEnd.3 cells treated with exogenous Aβ: pre-IP samples and samples immunoprecipitated with antibodies to pS8-Aβ ("1E4E11"). A photograph of nitrocellulose membrane after Western blotting, incubated with 1E4E11 antibodies, are shown.

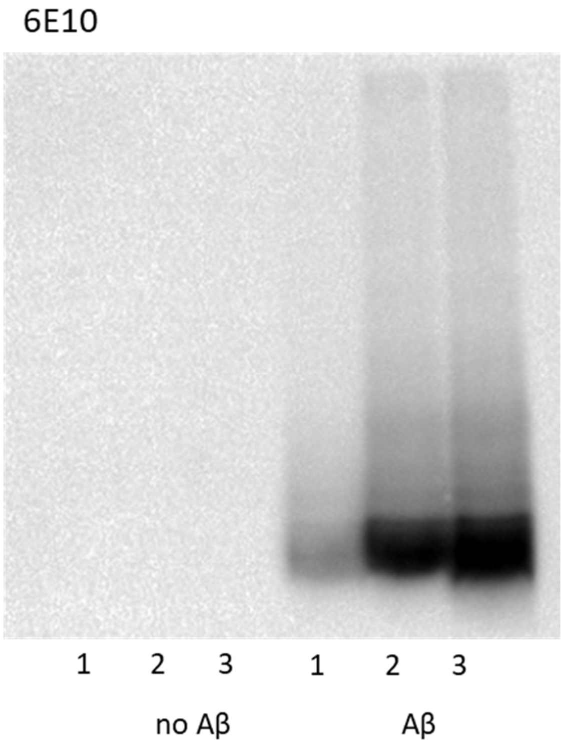

Figure S3. Total Aβ in cell lysate of bEnd.3 cells treated with exogenous Aβ (“Aβ”) or in control samples (“no Aβ”). A photograph of nitrocellulose membrane after Western blotting, incubated with antibodies to Aβ epitope 7-14 (“6E10”) are shown.

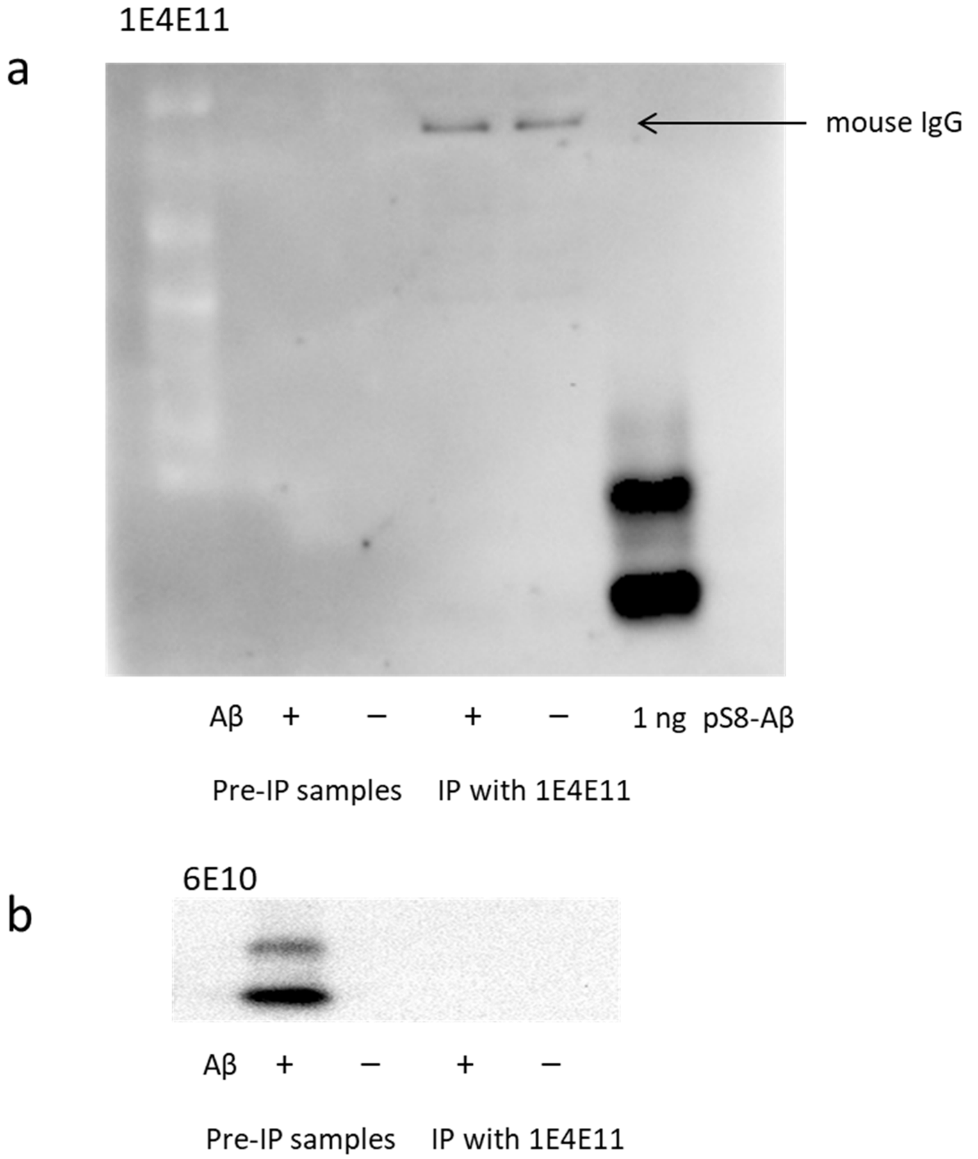

Figure S4. Phosphorylated Aβ in cell media of bEnd.3 cells treated with exogenous Aβ: pre-IP samples and samples immunoprecipitated with antibodies to pS8-Aβ ("1E4E11"). A photograph of nitrocellulose membrane after Western blotting, incubated with 1E4E11 antibodies (**a**) and with 6E10 antibodies (**b**), are shown.

Supplementary Methods

Measuring TBBz toxicity with WST test

HEK293 were plated on 96-well plates (Greiner) and grown till confluent. Dry TBBz (the CK2 inhibitor) from Sigma-Aldrich was dissolved in DMSO to 11 mM and stored at -20 in aliquots. An aliquot was stepwise diluted in DMEM to 1, 10 or 25 μM. The media in wells was replaced with DMEM containing TBBz or no TBBz (control). Cells were incubated for 6 or 24 hours in CO_2 incubator. Then, cell viability was measured with WST kit (CELLPRO-RO, Merck) according to manufacturer's protocol.
